# Local Probiotic *Lactobacillus crispatus* and *Lactobacillus delbrueckii* Exhibit Strong Antifungal Effects Against Vulvovaginal Candidiasis in a Rat Model

**DOI:** 10.3389/fmicb.2019.01033

**Published:** 2019-05-08

**Authors:** Ting Li, Zhaohui Liu, Xu Zhang, Xi Chen, Suxia Wang

**Affiliations:** ^1^Department of Gynecology, Beijing Obstetrics and Gynecology Hospital, Capital Medical University, Beijing, China; ^2^Ultrastructural Pathology Center, Peking University First Hospital, Beijing, China; ^3^Laboratory of Electron Microscopy, Department of Gynecology, Minimally Invasive Center, Beijing Obstetrics and Gynecology Hospital, Capital Medical University, Beijing, China

**Keywords:** *Lactobacillus crispatus*, *Lactobacillus delbrueckii*, vulvovaginal candidiasis, antifungal, microbiome

## Abstract

A comprehensive knowledge of the vaginal ecosystem is critical for the development of successful approaches to the treatment of infections. The role of *Lactobacilli* in preventing vulvovaginal candidiasis (VVC) is controversial. In this study, we investigated the therapeutic effects and mechanism of *Lactobacillus crispatus* or *delbrueckii* on vaginitis caused by *Candida albicans* in a Sprague–Dawley rat model. A microbiological evaluation was performed by Gram staining and fungal colonies were enumerated. The antifungal efficacy of the two *Lactobacillus* strains was assessed by hematoxylin and eosin (HE) staining, transmission electron microscopy (TEM), immunohistochemical detection of interferon-γ (IFN-γ), interleukin (IL)-4, IL-17, and epithelial-derived IgG (RP125). Our *in vitro* results showed that the inhibitory activity against *Candida* colony-forming unit (CFU) counts was demonstrated by the two *Lactobacillus* strains (*P* < 0.001). Our results indicated that *Lactobacillus* administration played an indispensable role in maintaining the immune homeostasis, and decreasing the Th1/Th2 ratio (IFN-γ/IL-4) by regulating the epithelial secretion of cytokines that inhibit epithelial proinflammatory cytokine release, while increasing epithelial-derived IgG expression (*P* < 0.05), suggesting antibody-mediated protection. Our results implicate *L. crispatus* and *L. delbrueckii* as a potential adjunct biotherapeutic agent in women with VVC, especially for those with drug resistance, adverse effects or contraindications when using antifungal agents. Further large, long-term, well-planned clinical studies should be performed in clinical practice to determine their clinical potential of *L. crispatus* and *L. delbrueckii* as an adjunct treatment for VVC.

## Introduction

It is now well accepted that the microbiota present in the human body can impact immunity, physiology, and health (Song et al., 2018). It has been postulated that the unique vaginal microbiome has evolved to perform the dual roles of disease resistance and obstetric protection (Amabebe and Anumba, 2018). Moreover, infections can arise from imbalances in the highly diverse vaginal microbiota (e.g., VVC). VVC, caused primarily by *Candida*, is the second-most common vaginal infecti and is associated with vulval discomfort or and pain (Dovnik et al., 2015). The recommended standard therapy for vaginal *C. albicans* infections is antifungal therapy, consisting of oral or intravaginal azole, or triazole drugs (Sobel and Sobel, 2018). However, prolonged use of antibiotic administration increases recurrence rates, probably due to an inability to re-establish the normal *Lactobacillus*-dominated vaginal flora.

In recent years there has been growing interest in the therapeutic use of probiotics. *Lactobacillus* species in general are recommended as a new strategy, which is increasing in popularity based on accumulating evidence for their effectiveness in restoring normal microbial function and preventing urogenital infections (Amabebe and Anumba, 2018). Probiotic use has been associated with a significant reduction in the recurrence of VVC (Palacios et al., 2016). First introduced in 1974 by Parker (Fuller, 1999), the word “probiotic” is used to describe live microorganisms that provide a benefit to the recipient when administered in sufficient quantities (World Health Organization and Food and Agriculture Organization of the United Nations, 2006).

The vaginal microbiota in most healthy women of reproductive-age is dominated by protective *Lactobacillus* species that are thought to reinforce the defense against invasion and colonization by pathogenic microorganisms (Melgaço et al., 2018). In women with vaginal infections, such as BV and VVC, the dominance of these *Lactobacilli* is compromised (Sobel, 2007). Consequently, intravaginal probiotics of the *Lactobacillus* genus are common and act beneficially in a number of ways. In addition to their function as a barrier to vaginal colonization by harmful microorganisms (Kim and Park, 2017), these species maintain an acidic intravaginal microflora by generating lactic acid via carbohydrate decomposition (Cadieux et al., 2002), inhibit catalase-negative anaerobic organisms by production of hydrogen peroxide (H_2_O_2_) as a source of free radicals (Lamont, 2003) and produce antimicrobial peptides, such as bacteriocin-like substances and biosurfactants (Witkin et al., 2013). In addition, *Lactobacillus* species induce anti-inflammatory immune responses in the host via the NF-κB pathway (Santos et al., 2018).

More than 20 species of *Lactobacilli* have been detected in the vagina, of which *L. crispatus* is the most prevalent in healthy Asian women and has been widely investigated as a vaginal probiotic due to its strong antimicrobial activity (Ravel et al., 2011).

*Lactobacillus crispatus* confers protection on the epithelial barrier against injury and inflammation by regulating epithelial cell function (Anton et al., 2018). Our previous study showed that *L. crispatus* attenuates the virulence of *C. albicans*, modulates the secretion of cytokines and chemokines, and enhances the immune response of VK2/E6E7 cells *in vitro* (Niu et al., 2017). However, there are no commercially available vaginal preparations containing *L. crispatus* in the Chinese market. Live *Lactobacillus* Capsule for Vaginal Use, containing *L. delbrueckii* (Neimenggu Wanzeshuangqi Pharmaceutical Co., Ltd.) is the only commercial preparation of *Lactobacillus* that is currently available. This preparation has been introduced to inhibit the cytotoxic effects and adhesion of pathogenic organisms (Banerjee et al., 2009). *L. delbrueckii* produces large amounts of H_2_O_2_ and has been reported to inhibit *C. albicans* more effectively than many other vaginally derived strains found in healthy women (Strus et al., 2005). Due to the close anatomic relationship with the vagina, the anus is convenient for the migration of gut organisms, including oral administration of probiotic *Lactobacillus* strains (e.g., yogurt drinks), to the vagina. Passive transfer of *Lactobacilli* to the vagina from the rectum may be an important consideration in the delivery of probiotics in foods and dietary supplements (Buggio et al., 2019). Intake of oral probiotics containing *Lactobacillus* strains improved the recovery rate and symptoms of patients with vaginal infections and tended to improve the vaginal microbial pattern (Laue et al., 2018).

Considering the proven health promoting effects of *L. crispatus*, investigations of the efficacy of topical *Lactobacillus* strains on treatment of VVC are warranted. To date, few, if any, studies have assessed the anti-*Candida* activities and probiotic properties of *Lactobacillus* strains in VVC. Therefore, the objective of this study was to investigate and compare the effects of (1) *L. crispatus* as the predominant vaginal bacteria in humans, and (2) *L. delbrueckii*, which is contained in the only commercially available vaginal preparation of *Lactobacillus* strains, to prevent the invasion of *C. albicans*, additionally focusing on the immune response of the host vaginal epithelium in a rat model.

## Materials and Methods

### Microorganism and Growth Conditions

*Lactobacillus crispatus* [American Type Culture Collection (ATCC) 33820] was routinely cultured anaerobically at 37°C in de Man, Rogosa and Sharpe broth (Becton Dickinson, Cockeysville, MD, United States). After overnight culture, cells were collected by centrifugation (10,000 × *g*, 10 min, 5°C), washed twice, and resuspended in 0.9% saline solution at 3 × 10^8^ CFU/ml. The vaginal *Lactobacillus* (Dingjunsheng^®^; Neimenggu Wanzeshuangqi Pharmaceutical Co., Ltd.) probiotic preparation consisted of 0.25 g capsules containing approximately 0.25 × 10^6^ CFU of live *L. delbrueckii*. Suspensions were formulated daily in saline solution to achieve approximately 3 × 10^8^ CFU/ml. *L. crispatus* or *L. delbrueckii* suspensions were administered intravaginally to each animal once daily at 3 × 10^8^ CFU/ml.

*Candida albicans* strains (ATCC-11006) were cultured to the third generation on Sabouraud dextrose agar (SDA; Becton Dickinson) at 37°C for 72 h and harvested. After resuspension in 0.9% saline solution, the cells were counted using a hemocytometer (Hausser Scientific; Horsham, PA, United States) and adjusted to a final concentration of 1 × 10^5^ CFU/ml.

### Animals

Female Sprague–Dawley rats (*n* = 24, 210–240 g), obtained from Beijing Vital River Laboratory Animal Technology Co. Ltd. (Beijing, China), were maintained in cages under a 12-h light/ 12-h dark cycle with free access to commercial food and water.

### Experimental Candidal Vaginitis

**FIGURE 1:**
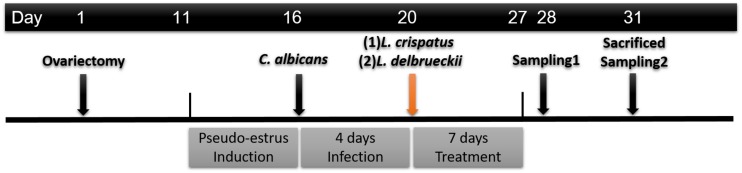
Timeline of the experimental animal treatment and sample collection.

The timeline of the experimental animal treatment and sample collection is shown in Figure 1. Prior to infection, animals received subcutaneous injection of 0.5 mg estradiol benzoate (Estradiolo, Amsa Farmaceutici, Rome, Italy) at 2-day intervals until the end of the experiment to induce immunosuppression (Zhang et al., 2018). The hormonal activity was assessed by analysis of the enucleated epithelial cells in vaginal fluid smears verified under a light microscope; enucleated cornified cells indicated the pseudo-estrus phase (Mandl, 1951). The rats were inoculated intravaginally with a yeast suspension of 1 × 10^8^ yeasts/mL (in 150 mL of sterile saline solution) of washed *C. albicans* blastoconidia. Four days after inoculation but before treatment, samples were obtained from the vaginal cavity to confirm *C. albicans* infection by Gram staining of the yeast/hyphae-like form and vaginal cells observed under light microscopy.

The rats were randomly divided into four groups (*n* = 6 rats per group). At 24 h after the initial infection, rats were randomly divided into following four groups (*n* = 6 rats per group); (a) uninfected (Control, vaginal inoculation with 0.9% saline solution); (b) infected (Model, vaginal inoculation with 0.9% saline solution); (c) infected but received intravaginal *L. crispatus* (approximately 3.0 × 10^8^ CFU/mL) once a day for 7 days; and (d) infected but received intravaginal *L. delbrueckii* (approximately 3.0 × 10^8^ CFU/mL) once a day for 7 days. At 1 (Day 28) and 4 days (Day 31) after the 7-day treatment period, a microbiological evaluation was performed by Gram staining. The *C. albicans* load in vaginal lavage (two sequential 500 μl volumes of sterile saline) was determined by the CFU assay on SDA. The antifungal effects were analyzed by enumerating the CFU/mL within the samples. All rats were anesthetized with an intraperitoneal injection of 100 mg/kg of ketamine hydrochloride and euthanized. Whole-vagina samples were harvested.

### Histological Analysis

Following immersion in formalin, vaginal samples were cut into 4-μm thick sections and stained using a HE staining kit (Beyotime Biotechnology, Beijing, China) according to the manufacturer’s instructions. The tissues were then dehydrated by immersion in a gradient alcohol series followed by xylene clearing. After sealing with a neutral balsam, the tissues were evaluated for pathological changes under a microscope (Olympus).

### Immunohistochemistry

Sections were incubated overnight at 4°C with the following primary detection antibodies: anti-rat-IFN-γ (1:100), anti-rat-IL-4 (1:200), anti-rat-IL-17 (1:50) (all rabbit polyclonal, Cloud-Clone, United States), and anti-IgG (RP215, provided by the Immunology Department, Peking University Health Science Center; 1:100). Sections were then incubated with horseradish peroxidase-conjugated anti-rabbit Ig (Zhongshan Golden Bridge Biotechnology) and immunoreactivity was developed using the 3,3′-diaminobenzidine (DAB) substrate system. Negative control sections were incubated with PBS instead of the primary antibody. Semi-quantitative analysis was performed by an experienced pathologist based on IRS (Dereci et al., 2017) calculated by multiplying SP scores: 0, <10%; 1, 10–25%; 2, 26–50%; 3, 51–75%; 4, 76–100%) and SI scores: 0, negative; 1, mild; 2, moderate; 3, severe).

### Transmission Electron Microscopy (TEM) Examination

Vaginal tissue pieces were removed, fixed (3% glutaraldehyde and 1% osmium tetroxide), dehydrated in a graded ethanol series and embedded in a PON812 resin (SPI, West Chester, PA, United States). Tissues were. then stained with 5% uranyl acetate and lead citrate. Ultrastructural changes in the vaginal tissues were observed under a JEM 1230 TEM (JEOL Co., Hitachi Ltd., Tokyo, Japan) and photographed.

Desmosomes within the epithelium were identified and enumerated by two independent investigators according to a previously described method (Capocelli et al., 2015). Desmosomes were identified and quantified as discrete, linear hyper-densities along the outer cellular membrane that confer structural integrity to tissues. Additional morphological features of the epithelia were identified independently by two investigators.

### Statistical Analysis

All data were analyzed using statistical analysis software SPSS 13.0. Data represent the mean ± standard deviation (*n* = 6 animals per group) of three independent experiments. Quantitative variables were tested for normal distribution and data were compared using a single-factor analysis of variance (ANOVA) using Windows software (SPSS Inc., Chicago, IL, United States). Fisher’s LSD test was used to evaluate differences between two groups. *P* < 0.05 was considered to indicate statistical significance.

## Results

### *L. crispatus* and *L. delbrueckii* Protect Against *C. albicans* in VVC Rats

**FIGURE 2:**
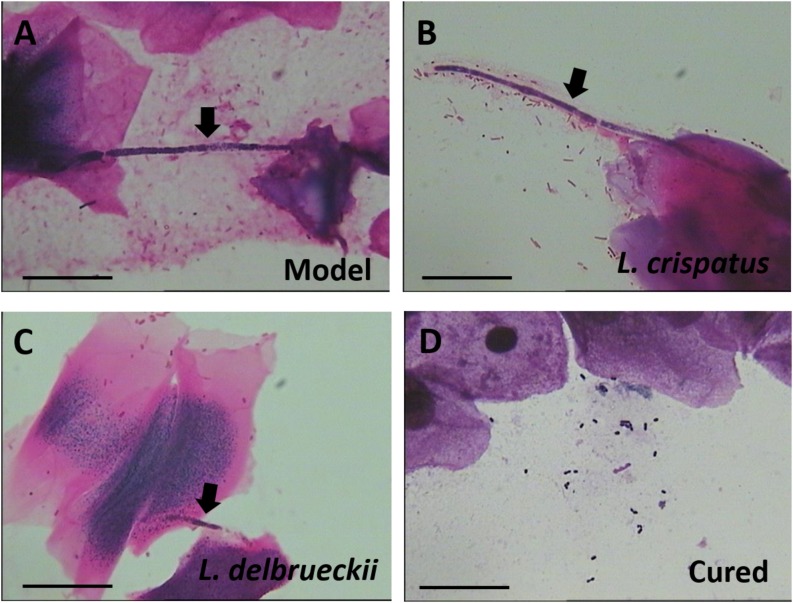
Gram staining of a vaginal swab from Model **(A)** and *Lactobacilli*-treated rats **(B–D)**. *Candida albicans* hypha (black arrow) adhering to vaginal epithelial cells. The probiotic *Lactobacilli* surround and inhibit the growth of *C. albicans* hyphae. Scale bar = 20 μm, magnification × 1,000. Model (without treatment); *L. crispatus* treated (3.0 × 10^8^ CFU for 7 days, Gram stain-positive); *L. delbrueckii* treated (3.0 × 10^8^ CFU for 7 days, Gram stain-positive); and Cured (3.0 × 10^8^ CFU of *L. crispatus* for 7 days, Gram stain-negative).

To compare the antifungal effects of *L. crispatus* and *L. delbrueckii*, rats were infected intravaginally with the mucosal *C. albicans*. At 1 and 4 days after the last dose, samples were obtained from all animals, and assessed by Gram staining. The results showed that vaginal administration of *L. crispatus* or *L. delbrueckii* exerted similarly, weak anti-*C. albicans* vaginitis effects. Among the untreated Model rats that remained infected, all vaginal swabs tested positive for *C. albicans*, with the epithelial cells being penetrated by the hypha (Day 31, Figure 2A). However, two of the six rats with positive vaginal swabs were Gram stain-negative after receiving intravaginal *L. crispatus* or *L. delbrueckii* (Day 31), suggesting less than half of the infected rats were cured. The negative pathogen conversion rate of *L. crispatus* at 1 (Day 28) and 4 days (Day 31) after treatment reached 33.3% (2/6), and 33.3% (2/6), respectively, and there were no significant differences compared with the negative pathogen conversion rate of the corresponding *L. delbrueckii* groups [0% (0/6) at 1 day (Day 28) and 33.3% at 4 days (Day 31) after treatment; *P >* 0.05; Figures 2B–D].

**FIGURE 3:**
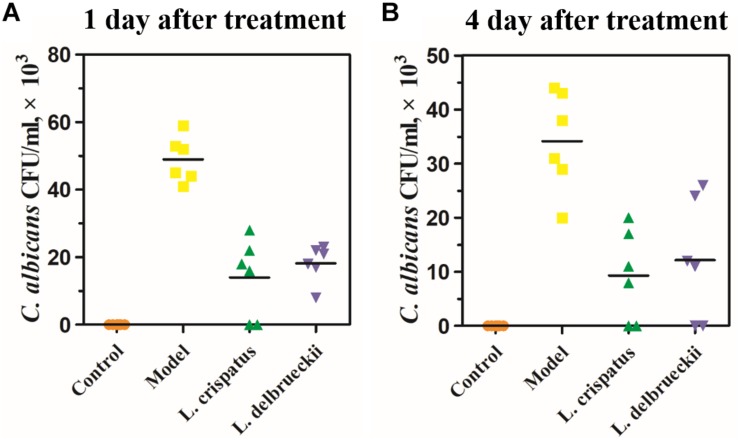
The number of colony-forming units (CFU)/mL of samples of vaginal tissue obtained from rats (**n** = 6 rats/group) at 1 day **(A)** and 4 days **(B)** after treatment and incubated on Sabouraud dextrose agar. Uninfected female Sprague-Dawley rats were used as controls. Model rats were infected with 1 × 10^8^ CFU/ml **C. albicans**. Control (uninfected); Model: infected with 1 × 10^8^ CFU/ml **C. albicans**;**L. crispatus** treated: infected and treated with **L. crispatus** 3.0 × 10^8^ CFU for 7 days; **L. delbrueckii**treated: infected and treated with **L. delbrueckii** 3.0 × 10^8^ CFU for 7 days.

Next, we assessed the antifungal effects of intravaginal *L. crispatus* or *L. delbrueckii* on the fungal burden by quantifying the colony formation (Figure 3). At baseline, Control rats were free of *C. albicans*. The mean CFU/mL for *C. albicans* at Day 28 was 49.00 ± 2.77 × 10^3^ in the Model group at Day 28, and was followed by a slow decline in vaginal *Candida* burden to 34.17 ± 3.78 × 10^3^ at Day 31. One day after the treatment (Day 28), the mean CFUs in the *L. crispatus* (14.00 ± 4.73 × 10^3^) and *L. delbrueckii* (18.17 ± 2.24 × 10^3^) groups were significantly lower than those in the Model group (Figure 3A and Table 1; *P* < 0.001). Four days after the treatment (Day 31), the mean CFUs in the *L. crispatus* (9.33 ± 3.42 × 10^3^), and *L. delbrueckii* (12.17 ± 4.58 × 10^3^) groups were significantly lower than those the Model group (Figure 3B and Table 1; *P* < 0.001). There were no significant differences in the mean CFUs in the *L. crispatus*, and *L. delbrueckii* groups at one (Day 28) or 4 day (Day 31) after the treatment (*P >* 0.05).

**TABLE 1:** Number of uninfected animals and quantification of fungal burden.

Treatment groups		Control	Model	*L. crispatus*	*L. delbrueckii*	*P*-value
Day 1 after treatment (Day 28)	CFU/mL (×10^3^)	0.00 ± 0.00	49.00 ± 2.77	14.00 ± 4.73	18.17 ± 2.24	<0.001
	Uninfected rats (N/n)^a^	6/6	0/6	2/6	0/6	
Day 4 after treatment (Day 31)	CFU/mL (×10^3^)	0.00 ± 0.00	34.17 ± 3.78	9.33 ± 3.42	12.17 ± 4.58	<0.001
	Uninfected rats (N/n)	6/6	0/6	2/6	2/6	

*^a^N, number of uninfected animals/n: total animals per group.*

### Morphometric Analysis

**FIGURE 4:**
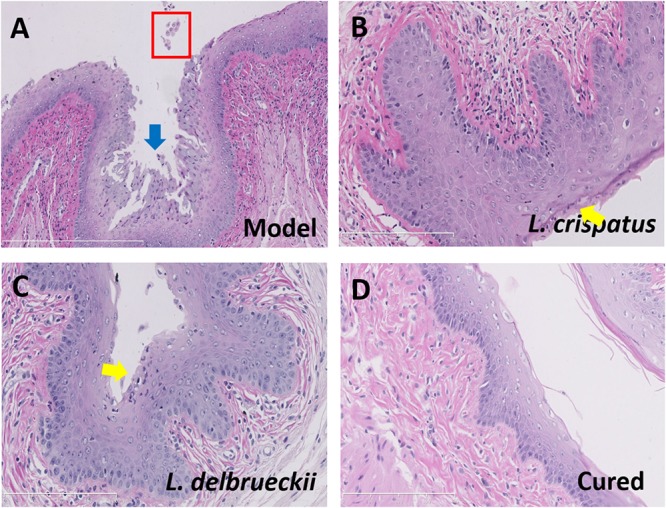
Histology of vaginal tissue sections from Model **(A)** and **Lactobacilli**-treated rats **(B–D)**, stained with hematoxylin-eosin. Damaged vaginal epithelium (blue arrow), exfoliated vaginal epithelial cells (red box), and neutrophils (yellow arrow) infiltrating the vaginal epithelium are shown. Scale bar = 50 μm. Model (without treatment);**L. crispatus**-treated (3.0 × 10^8^ CFU for 7 days, with neutrophil infiltration); **L. delbrueckii**-treated (3.0 × 10^8^ CFU for 7 days, with neutrophil infiltration); and Cured (3.0 × 10^8^ CFU of **L. crispatus** for 7 day, without neutrophil infiltration).

We performed HE staining and TEM analyses of anatomical and ultrastructural changes to evaluate the probiotic properties of the two *Lactobacillus* strains. The epithelium and mucosa in uninfected Control animals appeared normal, with no signs of inflammation, and a thick layer of stratum corneum was observed in the vaginal canal. All Model animals presented abnormal vaginal discharge and tissue swelling, as well as severe inflammation of the epithelium following *Candida* infection. HE staining revealed serious damage to the vaginal mucosa, with epithelial cell exfoliation and inflammatory cell infiltration, following infection with *C. albicans* (Figure 4A). Compared to the Model animals mucosa, the animals treated with *L. crispatus* or *L. delbrueckii* for 7 days showed a significant alleviation of inflammation and damage to the vaginal epithelial mucosa. In addition, an almost complete restoration of the mucosa was observed although some inflammatory cells persisted on the surface of the mucosa and in the lamina propria (Figures 4B,C). Several damaged tissues were observed with a complete restoration of a healthy vaginal mucosa (Figure 4D).

### *L. crispatus* and *L. delbrueckii* Promote the Integrity of the Ultrastructural Morphology of the Vaginal Mucosal Barrier

**FIGURE 5:**
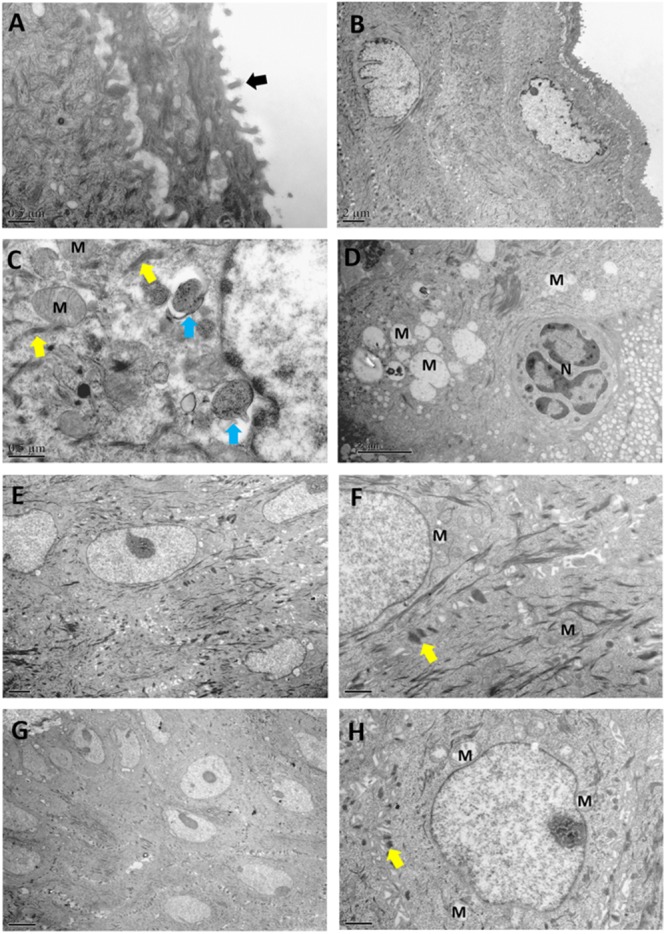
Ultrastructural changes in the vaginal mucosa of rats observed by transmission electron microscopy. Control, uninfected **(A,B)**; Model, infected with 1 × 10^8^ CFU/ml *C. albicans* **(C,D)**; *L. crispatus* treated, infected and treated with *L. crispatus* 3.0 × 10^8^ CFU for 7 days **(E,F)**; *L. delbrueckii* treated, infected and treated with *L. delbrueckii* 3.0 × 10^8^ CFU for 7 days **(G,H)**. Rats in the Control group show normal vaginal morphology with a non-keratinized stratified squamous epithelium and intact microvilli (black arrow) extended by vaginal epithelial cells. Yeast cells (blue arrow) invading the superficial layers of the vaginal mucosa, with injured desmosome-like junctions (yellow arrow) and swollen mitochondria (M) after infection with 1 × 10^8^ CFU/ml *C. albicans*.

**FIGURE 6:**
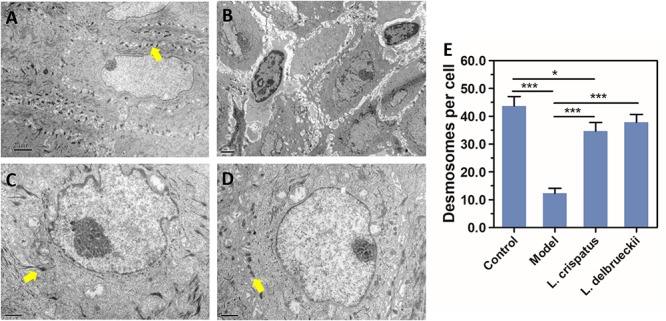
Desmosome quantification by TEM. Ultrastructure of desmosomes in the vaginal epithelium of rats observed by TEM. Control, uninfected **(A)**; Model, infected with 1 × 10^8^ CFU/ml **C. albicans** **(B)**;**L. crispatus** treated, infected and treated with **L. crispatus** 3.0 × 10^8^ CFU for 7 days **(C)**; **L. delbrueckii**treated, infected and treated with **L. delbrueckii** 3.0 × 10^8^ CFU for 7 days **(D)**. The desmosome-like junctions (yellow arrow) are significantly diminished, and the space between adjoining epithelial cells is widened in Model rats. Number of desmosomes per cell **(E)** in normal Control, Model, **L. crispatus** treated and **L. delbrueckii**treated rats. Data represent the mean ± standard deviation (**n** = 6). *P < 0.05; **P < 0.01; ***P < 0.001.

Under TEM observation, the normal vaginal mucosal barrier consists of tightly packed stratified squamous non-keratinized epithelial cells (i.e., desmosomes, as rigid plaques that maintain tissue integrity and intercellular adhesion) with numerous intact finger-like and/or bubble-like pseudopods or microvilli on their surface (Figures 5A,B). The usual structure of desmosomes is clearly identified by the presence of outer and inner dense plaques on the cytoplasmic side of the plasma membrane. After *C. albicans* infection, neutrophils and spores infiltrating the vaginal epithelium and lamina propria were observed. In accordance with a previous study (Harmon and Green, 2013), histological evaluation showed that the intracellular spaces were enlarged, desmosomal junctions were significantly impaired and blurred, and cytoplasmic vacuolization was present in non-keratinocytes and intercellular disjunctions (Figures 5C,D). The number of desmosomes per cell was significantly reduced in epithelia after challenge compared with that in normal epithelia (43.67 ± 8.43 vs. 12.33 ± 4.32, *P* < 0.0001). Intracellularly, mitochondria exhibited swelling, as well as crest deformation and dissolution. Furthermore, compared with the Model group, we observed a marked ultrastructural improvement in the vaginal epithelium following treatment with either of the *Lactobacillus* strains, with significant reductions in the numbers of adhesive yeast, desmosome-like junctions and hyphal forms. The morphology of the mitochondria was normal or showed only slight swelling in the *L. crispatus* (Figures 5E,F) or *L. delbrueckii* groups (Figures 5G,H) when compared with that in the Model groups. The number of desmosomes in the *L. crispatus* group was significantly increased compared with that in the Model group (34.67 ± 7.61, *P* < 0.0001; Figure 6). Moreover, the number of desmosomes in the *L. delbrueckii* group (37.83 ± 6.97) was almost the same as that observed in normal epithelia (Figure 6; *P* = 0.443). These observations indicated that *Lactobacillus* induced a partial restoration of the mucosa.

### *L. crispatus* and *L. delbrueckii* Modulate Cytokine Release by Vaginal Epithelial Tissue

**FIGURE 7:**
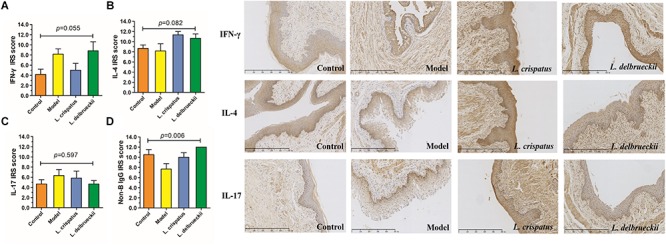
The expression of IFN-γ, IL-4 and IL-17 in vaginal tissues. Semi-quantitative analysis by immunoreactivity score (IRS) of IFN-γ, IL- 4 and IL-17 detected by immunohistochemistry. Control, uninfected **(A)**; Model, infected with 1 × 10^8^ CFU/ml *C. albicans* **(B)**; *L. crispatus* treated, infected and treated with *L. crispatus* 3.0 × 10^8^ CFU for 7 days **(C)**; *L. delbrueckii* treated, infected and treated with *L. delbrueckii* 3.0 × 10^8^ CFU for 7 days **(D)**. Data represent the mean ± standard deviation (*n* = 6). Scale bar = 50 μm.

Our immunohistochemical studies revealed increased expression of IFN-γ (IRS: 4.17 ± 2.57 vs. 8.17 ± 2.57) and IL-17 (IRS: 4.67 ± 2.07 vs. 6.33 ± 2.88), indicating stimulation of Th 1 and Th 17-type immune responses in the vaginal epithelium following *C. albicans* infection. However, there were no significant differences in the IRS scores for IFN-γ (*P* = 0.055, Figure 7A), IL-4 (*P* = 0.082, Figure 7B), and IL-17 (*P* = 0.017, Figure 7C) among the groups. The IFN-γ/IL-4 ratio was calculated to reflect the Th1/Th2 balance (Table 2). When challenged with *C. albicans*, the IFN-γ/IL-4 ratio was significantly higher than that of the untreated animals (0.47 ± 0.24 vs. 1.26 ± 0.87, *P* = 0.012). In contrast, the ratio was significantly lower in the *L. crispatus* group following *in vivo* challenge (0.43 ± 0.26, *P* = 0.009) compared than that in the *L. delbrueckii* group (0.80 ± 0.36, *P* = 0.219), for which the ratio was similar to that of the normal tissue (*P >* 0.05).

**TABLE 2:** Immunoreactivity score (IRS) of Th1/Th2 ratio (IFN-γ/IL-4) (*n* = 6, ANOVA).

Th1/Th2	IRS scores	*F*-value	*P*-value
Control	0.47 ± 0.24^a^	3.563	0.033
Model	1.26 ± 0.87		
*L. crispatus*	0.43 ± 0.26^a^		
*L. delbrueckii*	0.80 ± 0.36		

*^a^*P* < 0.05 compared to Model.*

Although the vaginal mucosa acts a barrier against pathogenic invasion, there is no direct evidence that vaginally expressed Ig molecules contribute to antimicrobial activity. The monoclonal antibody RP215 binds specifically to a unique glycosylated epitope on non-B cell, epithelial-derived IgG. Strong R215-positive staining was detected in the cytoplasm of normal vaginal epithelial cells, while staining in the submucosa tissue was weak. There were significant differences in the IRS scores for epithelial-derived IgG among the groups (*P* = 0.006, Figure 7D). The IRS score for epithelial IgG secreted by the vaginal mucosa was 10.50 ± 2.51, which dropped sharply following infection (6.67 ± 3.27; *P* = 0.010). However, when treated with *L. crispatus*, the score was significantly increased (10.00 ± 2.19, *P* = 0.022) with a similar pattern observed in the *L. delbrueckii* group (12.00 ± 0.00, *P* = 0.153), which reached the baseline values (*P >* 0.05).

## Discussion

*Lactobacilli* are Gram-positive rod-shaped, lactic acid producing bacteria which are mostly obligate and facultative anaerobes and predominantly found in the human genitourinary tract (Gupta et al., 1998). The *Lactobacilli* present in the vagina are important in preventing vaginal infections, such as BV and VVC, and invasion by pathogenic microorganisms, including human papillomavirus, and human immunodeficiency virus (Reid and Bocking, 2003). The currently available antimicrobial treatments for vaginal infections are associated with incomplete effectiveness leading to recurrent infections, drug resistance, and side-effects; therefore, alternative drugs are urgently required. In recent years, the use of *Lactobacilli*-derived probiotic bacteria has emerged as a novel strategy for the management of vaginal infections. However, investigations of the antifungal activities of probiotic strains are less common than investigations of their antibacterial activities (Chew et al., 2015). We have previously demonstrated that probiotic *Lactobacilli* can attenuate the virulence of *C. albicans* by modulating the secretion of cytokines and chemokines and enhancing the innate immune response *in vitro* (Niu et al., 2017). The functional characteristics of probiotics should be established based on both *in vitro* and *in vivo* studies. In the present study, intravaginal delivery of *Lactobacillus* strains for 7 days inhibited the *in vivo* growth of *Candida* species by between 60 and 70%. Thus, this animal model provides clear evidence of the fungicidal or candidacidal effects of the two probiotic *Lactobacillus* strains. Lopes et al. (2017) reported that *L. delbrueckii* showed no antimicrobial activity against pathogens, mainly due to the absence of organic acid production. However, in the present study, the antifungal effect of *L. delbrueckii* was similar to that of *L. crispatus*, which is believed to contribute to the control of vaginal microbiota by competing with other microflora for colonization of the vaginal epithelial cells (McLean and Rosenstein, 2000), as well as liberating H_2_O_2_ and other substances that mediate intracellular antifungal activity (Strus et al., 2006). These findings indicate the potential of these two probiotic *Lactobacillus* strains as an adjuvant to the mainstay in clinical treatment of VVC. However, the antifungal activity of probiotic *Lactobacillus* strains is very complex and requires further investigation (Ogunshe et al., 2011).

Maintaining the vaginal epithelial barrier is crucial as it maintains the balance between commensal microorganisms and the host. One of the potential health benefits of probiotics is the suppression of inflammatory responses (Isolauri et al., 2002). In our present study, upregulation of proinflammatory cytokines by *C. albicans* infection suggested T cell proliferation (IFN-γ and IL-17) and T cell activation (Bäuerl et al., 2013), while *Lactobacillus* treatment induced a partial reduction in the Th1/Th2 ratio. These observations suggest that both strains counteract the molecular events leading to T cell activation, as has been shown previously for the probiotic strains *L. paracasei* (Bäuerl et al., 2013), *L. plantarum* (Bäuerl et al., 2013), and *L. casei* (Carol et al., 2006). The two *Lactobacillus* strains used in this study, and particularly *L. crispatus*, were able to alter the cytokine profiles of vaginal epithelial cells, thus redirecting the pattern of immunity to a regulatory or tolerant mode that balances the Th1/Th2 ratio, and suppresses the proinflammatory cytokine production (e.g., IL17 and IFN-γ), although this difference was not statistically significant, which is in accordance with previous reports (Wasilewska et al., 2019). Decreased production of IFN-γ, a typical Th1 cytokine, could be a very interesting probiotic feature (Bäuerl et al., 2013). Although crucial for innate and adaptive immunity against intracellular pathogens, a prolonged distinct Th1-bias with a persistently high Th1/Th2 ratio leads to cell-mediated cytotoxicity, and inflammatory responses (Barousse et al., 2004; Ouyang et al., 2008). Taken together, these findings further support the hypothesis that *Lactobacilli* play an important role in maintaining the immune homeostasis by regulating cytokine secretion by epithelial cells (Bäuerl et al., 2013), and in this respect, *L. crispatus* treatment was more effective than *L. delbrueckii*. The sensing of, and signaling induced by, *Lactobacilli* require further investigation. *Lactobacillus*-derived proteins (Yan and Polk, 2012) and lactic acid induced acidification may be immunomodulatory functions that suppress the IFN-γ response of immune cells related to the Janus kinase/STAT signaling pathway and inhibit of the production of cytokines that mediate innate immunity (Fischer et al., 2007).

Previous studies have demonstrated that functional epithelial-derived IgG (RP215 positive) is secreted by vaginal epithelial cells *in vitro*. This finding challenges the classical concept that B cells are the only source of immunoglobulins (Li et al., 2016). These immunoglobulins appear to participate in skin innate immunity (Jiang et al., 2015) and may serve as natural antibodies (apparently the most controversial branch of humoral immunity), which not only recognize and neutralize antigenic microbial products (Amabebe and Anumba, 2018), but also provide long-term immunological memory that improves standard azole therapy efficacy and prevents relapse (Khasbiullina and Bovin, 2015). Our animal study confirmed our hypothesis that non-B IgG is expressed in the vaginal epithelium, a response that appears to be diminished by mycotic infections, but can be restored by *Lactobacillus* treatment. Further studies are required to ascertain the molecular mechanism by which *Lactobacilli* participate in the mucosal immunity in the vagina related to epithelial-derived IgG.

Another potential health benefit of *Lactobacillus* treatment is provided by the enhanced epithelial cell survival and barrier function (Yan and Polk, 2012; Yan et al., 2017). Ultrastructural analysis by electron microscopy verified several key features of *in vivo* vaginal epithelial tissue, such as the presence of desmosomes and mitochondria. In epithelial tissues, cells are anchored to one another via junctional complexes e.g., desmosomes, to maintain tissue integrity, and protect against the external environment (Green and Gaudry, 2000). Infection with pathogenic *C. albicans* not only significantly stimulates inflammatory cells, but also induces significant ultrastructural changes in the host cells, such as inhibited desmosome assembly, eventually leading to reduced adhesive strength (Kowalczyk and Green, 2013). Desmosomes, one of the predominant types of adhesive intercellular junctions in vertebrate tissues, are challenged by a variety of factors such as toxins, pathogens and inflammatory factors, potentially leading to tissue damage (Celentano and Cirillo, 2017). The damage to the epithelia, which could be the consequence of the inflammatory process itself, is related to disruption of this barrier, and passage of pathogenic microorganisms or molecules, an effect that is positively affected by probiotics, which stabilize the barrier function. There are several reports related to the effects of probiotic *Lactobacilli* on the “recovery” of the vaginal mucosa.

*Lactobacilli* have been reported to decrease damage to the DNA of epithelial cells (Yeh et al., 2007), reduce epithelial permeability, and attenuate the inflammatory response (Ahrne and Hagslatt, 2011). *Lactobacilli* have also been reported to prevent the attachment of the pathogenic microorganism required to exert its effects on paracellular permeability through upregulating production of MUC3 mucin (Ahrne and Hagslatt, 2011) and ameliorate epithelial apoptosis by producing *Lactobacillus*-derived soluble proteins in an EGF receptor-dependent manner (Yan and Polk, 2012). Mitochondria are influenced by a multitude of vital signals involved in the regulation of energy metabolism and cellular survival. These signals promote or prevent cell survival by modulating mitochondrial function and structure (Sedlackova and Korolchuk, 2018). Fungal infections directly or indirectly impair mitochondrial ultrastructure, ultimately leading to destruction of the vaginal epithelium. However, in this study, we observed significant improvements in the ultrastructure of the vaginal epithelium and host cell mitochondria following the administration of *Lactobacilli*. *Lactobacilli* exert protective anti-oxidant effects by preventing mitochondrial-generated reactive oxygen species that induce membrane damage (Barbonetti et al., 2013).

This study provides evidence implicating *Lactobacilli* as a potential adjunct biotherapeutic agent in women with VVC, especially for those with drug resistance, adverse effects or contraindications when using antifungal agents. The effects of probiotic *L. crispatus* and *L. delbrueckii* in treating VVC are particularly noticeable in relation to changes to the vaginal mucosa that are microbiological, physiological and morphological in nature. In terms of antifungal, anti-inflammatory effects and mucosal repair, the effects of *L. crispatus* are similar to those of the commercial *L. delbrueckii* formulation. The promising results of this study highlighting the potential of *Lactobacillus* treatment of VVC in clinical practice require validation in well-designed clinical studies with larger sample sizes and long-term follow-up.

## Ethics Statement

All animal experiments were performed according to the National Institutes of Health Guide for the care and Use of Laboratory Animals. The protocol was approved by Peking University First Hospital Ethics Committee (Permit No. #J201634). All animals were housed in the Animal Center Laboratory of Peking University First Hospital.

## Author Contributions

ZL contributed to the main experimental conception and design. TL and XC performed the experiments. TL, XZ, and SW analyzed the data and contributed to reagents. TL wrote the manuscript. All authors approved the final version of the manuscript.

## Conflict of Interest Statement

The authors declare that the research was conducted in the absence of any commercial or financial relationships that could be construed as a potential conflict of interest.

## Abbreviations

ANOVA: one-way analysis of variance
ATCC: American type culture collection
BV: bacterial vaginosis
*C. albicans*: *Candida albicans*
CFU: colony-forming units
HE: hematoxylin and eosin
IFN: interferon
IL: interleukin
IRS: the immunoreactivity score
*L. crispatus*: *Lactobacillus crispatus*
LSD: least significant difference
MRS, de Man: Rogosa and Sharpe
SDA: sabouraud dextrose agar
SI: staining intensity
SP: staining percentage
TEM: transmission electron microscopy
Th: T helper
VVC: vulvovaginal candidiasis.

## References

[B1] Ahrne S. Hagslatt M. L. J. (2011). Effect of lactobacilli on paracellular permeability in the gut. *Nutrients* 3 104–117. 10.3390/nu3010104 22254077 PMC3257727

[B2] Amabebe E. Anumba D. O. C. (2018). The vaginal microenvironment: the physiologic role of *Lactobacilli*. *Front. Med.* 5:181. 10.3389/fmed.2018.00181 29951482 PMC6008313

[B3] Anton L. Sierra L. J. DeVine A. Barila G. Heiser L. Brown A. G. (2018). Common cervicovaginal microbial supernatants alter cervical epithelial function: mechanisms by which *Lactobacillus crispatus* contributes to cervical health. *Front. Microbiol.* 9:2181. 10.3389/fmicb.2018.02181 30349508 PMC6186799

[B4] Banerjee P. Merkel G. J. Bhunia A. K. (2009). *Lactobacillus delbrueckii ssp. bulgaricus* B-30892 can inhibit cytotoxic effects and adhesion of pathogenic Clostridium difficile to Caco-2 cells. *Gut. Pathog.* 1:8. 10.1186/1757-4749-1-8 19397787 PMC2680912

[B5] Barbonetti A. Vassallo M. R. Cinque B. Filipponi S. Mastromarino P. Cifone M. G. (2013). Soluble products of *Escherichia coli* induce mitochondrial dysfunction-related sperm membrane lipid peroxidation which is prevented by lactobacilli. *PLoS. One* 8:e83136. 10.1371/journal.pone.0083136 24358256 PMC3865092

[B6] Barousse M. M. Van Der Pol B. J. Fortenberry D. Orr D. Fidel P. L. Jr. (2004). Vaginal yeast colonisation, prevalence of vaginitis, and associated local immunity in adolescents. *Sex. Transm. Infect.* 80 48–53. 14755036 10.1136/sti.2002.003855 PMC1758371

[B7] Bäuerl C. Llopis M. Antolín M. Monedero V. Mata M. Zúñiga M. (2013). *Lactobacillus paracasei* and *Lactobacillus plantarum* strains downregulate proinflammatory genes in an ex vivo system of cultured human colonic mucosa. *Genes Nutr.* 8 165–180. 10.1007/s12263-012-0301-y 22669626 PMC3575885

[B8] Buggio L. Somigliana E. Borghi A. Vercellini P. (2019). Probiotics and vaginal microecology: fact or fancy? *BMC. Womens Health* 19:25. 10.1186/s12905-019-0723-4 30704451 PMC6357464

[B9] Cadieux P. Burton J. Gardiner G. Braunstein I. Bruce A. W. Kang C. Y. (2002). *Lactobacillus* strains and vaginal ecology. *JAMA* 287 1940–1941. 11960535 10.1001/jama.287.15.1940

[B10] Capocelli K. E. Fernando S. D. Menard-Katcher C. Furuta G. T. Masterson J. C. Wartchow E. P. (2015). Ultrastructural features of eosinophilic oesophagitis: impact of treatment on desmosomes. *J. Clin. Pathol.* 68 51–56. 10.1136/jclinpath-2014-202586 25359789 PMC4506753

[B11] Carol M. Borruel N. Antolin M. Llopis M. Casellas F. Guarner F. (2006). Modulation of apoptosis in intestinal lymphocytes by a probiotic bacteria in Crohn’s disease. *J. Leukoc. Biol.* 79 917–922. 10.1189/jlb.0405188 16641137

[B12] Celentano A. Cirillo N. (2017). Desmosomes in disease: a guide for clinicians. *Oral. Dis.* 23 157–167. 10.1111/odi.12527 27329525

[B13] Chew S. Y. Cheah Y. K. Seow H. F. Sandai D. Than L. T. (2015). Probiotic *Lactobacillus rhamnosus* GR-1 and *Lactobacillus reuteri* RC-14 exhibit strong antifungal effects against vulvovaginal candidiasis-causing *Candida glabrata* isolates. *J. Appl. Microbiol.* 118 1180–1190. 10.1111/jam.12772 25688886 PMC4406132

[B14] Dereci Ö. Akgün Ş. Celasun B. Öztürk A. Günhan Ö (2017). Histological evaluation of the possible transformation of peripheral giant cell granuloma and peripheral ossifying fibroma: a preliminary study. *Indian J. Pathol. Microbiol.* 60 15–20. 10.4103/0377-4929.200032 28195085

[B15] Dovnik A. Golle A. Novak D. Arko D. TakaČ I. (2015). Treatment of vulvovaginal candidiasis: a review of the literature. *Acta. Dermatovenerol. Alp. Pannonica Adriat.* 24 5–7. 25770305 10.15570/actaapa.2015.2

[B16] Fischer K. Hoffmann P. Voelkl S. Meidenbauer N. Ammer J. Edinger M. (2007). Inhibitory effect of tumor cell-derived lactic acid on human T cells. *Blood* 109 3812–3819. 17255361 10.1182/blood-2006-07-035972

[B17] Fuller R. (1999). “Probiotics for farm animals,” in *Probiotics. A Critical Review*, ed. Tannock G. W. (Wymondham: Horizon Scientific Press), 15–22.

[B18] Green K. J. Gaudry C. A. (2000). Are desmosomes more than tethers for intermediate filaments? *Nat. Rev. Mol. Cell Biol.* 1 208–216. 11252896 10.1038/35043032

[B19] Gupta K. Stapleton A. E. Hooton T. M. Roberts P. L. Fennell C. L. Stamm W. E. (1998). Inverse association of H_2_O_2_-producing lactobacilli and vaginal *Escherichia coli* colonization in women with recurrent urinary tract infections. *J. Infect. Dis.* 178 446–450. 10.1086/515635 9697725

[B20] Harmon R. M. Green K. J. (2013). Structural and functional diversity of desmosomes. *Cell. Commun. Adhes.* 20 171–187. 10.3109/15419061.2013.855204 24205984

[B21] Isolauri E. Rautava S. Kalliomäki M. Kirjavainen P. Salminen S. (2002). Probiotic research: learn from the evidence. *Allergy* 57 1076–1077. 12359008 10.1034/j.1398-9995.2002.23804.x

[B22] Jiang D. Ge J. Liao Q. Ma J. Liu Y. Huang J. (2015). IgG and IgA with potential microbial-binding activity are expressed by normal human skin epidermal cells. *Int. J. Mol. Sci.* 16 2574–2590. 10.3390/ijms16022574 25625513 PMC4346852

[B23] Khasbiullina N. R. Bovin N. V. (2015). Hypotheses of the origin of natural antibodies: a glycobiologist’s opinion. *Biochemistry* 80 820–835. 10.1134/S0006297915070032 26541997

[B24] Kim J. M. Park Y. J. (2017). Probiotics in the prevention and treatment of postmenopausal vaginal infections: review article. *J. Menopausal. Med.* 23 139–145. 10.6118/jmm.2017.23.3.139 29354612 PMC5770522

[B25] Kowalczyk A. P. Green K. J. (2013). Structure, function and regulation of desmosomes. *Prog. Mol. Biol. Transl. Sci.* 116 95–118. 10.1016/B978-0-12-394311-8.00005-4 23481192 PMC4336551

[B26] Lamont R. F. (2003). Infection in the prediction and antibiotics in the prevention of spontaneous preterm labour and preterm birth. *BJOG* 110 71–75. 12763116 10.1016/s1470-0328(03)00034-x

[B27] Laue C. Papazova E. Liesegang A. Pannenbeckers A. Arendarski P. Linnerth B. (2018). Effect of a yoghurt drink containing *Lactobacillus* strains on bacterial vaginosis in women - a double-blind, randomised, controlled clinical pilot trial. *Benef. Microb.* 9 35–50. 10.3920/BM2017.0018 29065710

[B28] Li T. Niu X. Zhang X. Wang S. Liu Z. (2016). Baofukang suppository promotes the repair of vaginal epithelial cells in response to *Candida albicans*. *AMB Exp.* 6:109. 10.1186/s13568-016-0281-1 27830496 PMC5102987

[B29] Lopes E. G. Moreira D. A. Gullón P. Gullón B. Cardelle-Cobas A. Tavaria F. K. (2017). Topical application of probiotics in skin: adhesion, antimicrobial and antibiofilm in vitro assays. *J. Appl. Microbiol.* 122 450–461. 10.1111/jam.13349 27862685

[B30] Mandl A. M. (1951). The phases of the estrous cycle in the adult white rat. *J. Exp. Biol.* 28 576–584. 6282572

[B31] McLean N. W. Rosenstein I. J. (2000). Characterisation and selection of a *Lactobacillus* species to re-colonise the vagina of women with recurrent bacterial vaginosis. *J. Med. Microbiol.* 49 543–552. 10.1099/0022-1317-49-6-543 10847208

[B32] Melgaço A. C. C. Blohem Pessoa W. F. Freire H. P. Evangelista de Almeida M. Santos Barbosa M. Passos Rezende R. T. (2018). Potential of maintaining a healthy vaginal environment by two *Lactobacillus* strains isolated from cocoa fermentation. *Biomed. Res. Int.* 2018:7571954. 10.1155/2018/7571954 30364031 PMC6186379

[B33] Niu X. X. Li T. Zhang X. Wang S. X. Liu Z. H. (2017). *Lactobacillus crispatus* modulates vaginal epithelial cell innate response to *Candida albicans*. *Chin. Med. J.* 130 273–279. 10.4103/0366-6999.198927 28139509 PMC5308008

[B34] Ogunshe A. A. Omotoso M. A. Bello V. B. (2011). The in vitro antimicrobial activities of metabolites from *Lactobacillus* strains on *Candida* species implicated in *Candida vaginitis*. *Malays. J. Med. Sci.* 18 13–25. 22589669 PMC3328930

[B35] Ouyang W. Chen S. Liu Z. Wu Y. Li J. (2008). Local Th1/Th2 cytokine expression in experimental murine vaginal candidiasis. *J. Huazhong. Univ. Sci. Technol. Med. Sci.* 2008 352–355. 10.1007/s11596-008-0329-9 18563341

[B36] Palacios S. Espadaler J. Fernández-Moya J. M. Prieto C. Salas N. (2016). Is it possible to prevent recurrent vulvovaginitis? The role of *Lactobacillus plantarum* I1001 (CECT7504). *Eur. J. Clin. Microbiol. Infect. Dis.* 35 1701–1708. 27393491 10.1007/s10096-016-2715-8 PMC5035666

[B37] Ravel J. Gajer P. Abdo Z. Schneider G. M. Koenig S. S. McCulle S. L. (2011). Vaginal microbiome of reproductive-age women. *Proc. Natl. Acad. Sci. U.S.A.* 108 4680–4687. 10.1073/pnas.1002611107 20534435 PMC3063603

[B38] Reid G. Bocking A. (2003). The potential for probiotics to prevent bacterial vaginosis and preterm labor. *Am. J. Obstet. Gynecol.* 189 1202–1208. 14586379 10.1067/s0002-9378(03)00495-2

[B39] Santos C. M. A. Pires M. C. V. Leão T. L. Silva A. K. S. Miranda L. S. Martins F. S. (2018). Anti-inflammatory effect of two *Lactobacillus* strains during infection with *Gardnerella vaginalis* and *Candida albicans* in a HeLa cell culture model. *Microbiology* 164 349–358. 10.1099/mic.0.000608 29458690

[B40] Sedlackova L. Korolchuk V. I. (2018). Mitochondrial quality control as a key determinant of cell survival. *Biochim. Biophys. Acta Mol. Cell. Res.* 1866 575–587. 10.1016/j.bbamcr.2018.12.012 30594496

[B41] Sobel J. D. (2007). Candidal vulvovaginitis. *Lancet* 369 1961–1971. 10.1016/S0140-6736(07)60917-9 17560449

[B42] Sobel J. D. Sobel R. (2018). Current treatment options for vulvovaginal candidiasis caused by azole-resistant Candida species. *Expert. Opin. Pharmacother.* 19 971–977. 10.1080/14656566.2018.1476490 29932786

[B43] Song J. Lang F. Zhao N. Guo Y. Zhang H. (2018). Vaginal lactobacilli induce differentiation of monocytic precursors toward langerhans-like cells: in vitro evidence. *Front. Immunol.* 9:2437. 10.3389/fimmu.2018.02437 30410487 PMC6211368

[B44] Strus M. Brzychczy-Włoch M. Gosiewski T. Kochan P. Heczko P. B. (2006). The in vitro effect of hydrogen peroxide on vaginal microbial communities. *FEMS Immunol. Med. Microbiol.* 48 56–63. 16965352 10.1111/j.1574-695X.2006.00120.x

[B45] Strus M. Brzychczy-Wloch M. Kucharska A. Gosiewski T. Heczko P. B. (2005). Inhibitory activity of vaginal *Lactobacillus bacteria* on yeasts causing vulvovaginal candidiasis. *Med. Dosw. Mikrobiol.* 57 7–17. 16130291

[B46] Wasilewska E. Zlotkowska D. Wroblewska B. (2019). Yogurt starter cultures of *Streptococcus thermophilus* and *Lactobacillus bulgaricus* ameliorate symptoms and modulate the immune response in a mouse model of dextran sulfate sodium-induced colitis. *J. Dairy Sci.* 102 37–53. 10.3168/jds.2018-14520 30343915

[B47] Witkin S. S. Mendes-Soares H. Linhares I. M. Jayaram A. Ledger W. J. Forney L. J. (2013). Influence of vaginal bacteria and D-and L-lactic acid isomers on vaginal extracellular matrix metalloproteinase inducer: implications for protection against upper genital tract infections. *mBio.* 4:e460-13. 10.1128/mBio.00460-13 23919998 PMC3735189

[B48] World Health Organization, and Food and Agriculture Organization of the United Nations. (2006). *Probiotics in Food: Health and Nutritional Properties and Guidelines for Evaluation.* Rome, IT: World Health Organization, Food and Agriculture Organization of the United Nations.

[B49] Yan F. Liu L. Cao H. Moore D. J. Washington M. K. Wang B. (2017). Neonatal colonization of mice with LGG promotes intestinal development and decreases susceptibility to colitis in adulthood. *Mucosal. Immunol.* 10 117–127. 10.1038/mi.2016.43 27095077 PMC5073052

[B50] Yan F. Polk D. B. (2012). Characterization of a probiotic-derived soluble protein which reveals a mechanism of preventive and treatment effects of probiotics on intestinal inflammatory diseases. *Gut. Microb.* 3 25–28. 10.4161/gmic.19245 22356855 PMC3337122

[B51] Yeh S. L. Lin M. S. Chen H. L. (2007). Inhibitory effects of a soluble dietary fiber from Amorphophallus konjac on cytotoxicity and DNA damage induced by fecal water in Caco-2 cells. *Planta. Med.* 73 1384–1388. 10.1055/s-2007-990228 17893827

[B52] Zhang X. Li T. Chen X. Wang S. Liu Z. (2018). Nystatin enhances the immune response against Candida albicans and protects the ultrastructure of the vaginal epithelium in a rat model of vulvovaginal candidiasis. *BMC Microbiol.* 18:166. 10.1186/s12866-018-1316-3 30359236 PMC6202846

